# Estimation of Time Since Death From Potassium Levels in Vitreous Humor in Cases of Unnatural Death: A Facility-Based Cross-Sectional Study

**DOI:** 10.7759/cureus.39572

**Published:** 2023-05-27

**Authors:** Sruthi S Kurup, Murugesa Bharathi, Gaurang Narayan, Vinayagamoorthi R, Rajesh R, Tarun Kumar Suvvari

**Affiliations:** 1 Forensic Medicine, Indira Gandhi Medical College and Research Institute, Puducherry, IND; 2 Obstetrics and Gynecology, Indira Gandhi Government Medical College and Hospital, Nagpur, IND; 3 Biochemistry, Indira Gandhi Medical College and Research Institute, Puducherry, IND; 4 Research, Squad Medicine and Research (SMR), Visakhapatnam, IND; 5 General Medicine, Rangaraya Medical College, Kakinada, IND

**Keywords:** post-mortem interval (pmi), vitreous humour, unnatural death, potassium, time since death

## Abstract

Background

Estimation of time since death/postmortem interval (PMI) forms a crucial component for all autopsy surgeons. With the subjectivity that is prevalent with conventional morphological and physical signs of death, newer-age modalities such as chemical analysis provide better precision. The easy accessibility and the resistance to putrefaction make vitreous humor the best choice for such chemical analysis. Thus, the present study's aim is to estimate the time since death in cases of unnatural death by investigating the potassium level changes in the vitreous humor.

Methodology

This is a facility-based cross-sectional study conducted in the mortuary under the Department of Forensic Medicine in a public tertiary healthcare teaching hospital in South India between August and September 2022. Deceased individuals fulfilling the inclusion and exclusion criteria were recruited in the study. Vitreous samples were collected from a single eye and analyzed using an autoanalyzer for potassium values. After due derivations, postmortem intervals were calculated from potassium values, then they were compared with the PMIs estimated using physical signs and those determined using official police records. Data were entered using MS Excel 20 (Microsoft Corporation, Redmond, Washington) and analyzed using the Statistical Package for the Social Sciences (SPSS) software, version 20 (IBM Corp., Armonk, NY).

Results

Of the 100 deceased individuals included in the study, 68% were men, and the majority (24%) belonged to the age group of 53-62 years. A linear relationship is said to exist between vitreous potassium concentration and postmortem interval. No correlation was seen between the ambient temperature and the potassium levels of the vitreous humor. PMI confirmed by the potassium levels seconded the PMI given by the police records and physical signs (Rigor mortis) (Spearman’s rho was statistically significant at the two-tailed level or at the 0.01 level with a kappa value of 0.88).

Conclusion

Potassium measurements in the vitreous humor to estimate the PMI are associated with improved accuracy and precision in determining the time since death. They are not affected by external factors, making them a reliable marker for the same.

## Introduction

When the death of a person is attributed to uncertain causes, an autopsy is a tool that comes to the rescue to establish an effective diagnosis [1]. In autopsy, estimation of the time since death (TSD)/postmortem interval (PMI) becomes important due to its role in determining appropriate civil and criminal repercussions. It is merely the calculation of a quantifiable and computable date and time frame along a time-dependent curve expressed within an interval range [2]. Autopsy surgeons commonly depend on the physical signs of death, which have much subjectivity. Thus, the reliability and accuracy of the PMI are jeopardized [3]. For all practical purposes, any death not resulting from an underlying disease can fall under the ambit of unnatural death. Unnatural deaths are conventionally a combination of multiple actions. Thus, estimating the TSD in such cases becomes extremely cumbersome, and the tradition of morphological diagnostics would fail in precision [4-7].

TSD is influenced and affected by various internal and external factors, such as the presence of a preexisting disease, the ambient environment determining the humidity and temperature, and the thermodynamics of the last terminal action. In such cases, newer-age methodologies such as chemical analysis aid in the determination of TSD [8,9]. During the agonal stages of death, many biochemical changes start to occur. These are typically ascribed to decreased levels of oxygen in the blood, changes in enzyme reactions, and a halt in the creation of metabolites. All physiological fluids exhibit similar variations, and estimates of these metabolite profiles can aid in calculating the passing of time [10,11].

One such common body fluid is vitreous humor. The common electrolytes effectively studied for estimating the TSD, predominantly in cases of natural death, include potassium, hypoxanthine, and lactic acid [12-15]. Post-death, there arises a steady increase in the concentration of potassium in the vitreous, which is attributed to the autolysis of the vascular choroidal and retinal cells of the eye [14,15]. Considering the easy accessibility and the resistance of vitreous humor to putrefaction, in the present study, we aimed to investigate the potassium electrolyte changes that occur in the vitreous humor and study their effect on estimating the PMI in cases of unnatural death.

## Materials and methods

This was a facility-based cross-sectional study conducted in the mortuary under the Department of Forensic Medicine in a public tertiary healthcare teaching hospital in South India between August and September 2022. After obtaining Institutional Research Committee (IRC) and Institutional Ethics Committee (IEC) approval (417/IEC-35/IGMC&RI/PP-22/2022), the subjects were recruited. A separate written informed consent was not obtained from the patient party because the ethical committee permitted an exemption for obtaining informed consent, considering the fact that any procedure pertaining to postmortem would include proper estimation of TSD, and any such investigation is a part of routine postmortem examination.

All cases brought as sudden death or unnatural death (death not attributed to any underlying disease) in whom postmortem was performed in the mortuary affiliated with the Department of Forensic Medicine of the designated setting were included in the study. Exclusion criteria comprised cases with a previous history of eye or orbital injury or ocular surgeries, posterior segment diseases, cases of unnatural death with charred/skeletonized bodies, bodies retrieved in extreme changes of decompositions, and pediatric cases (less than 12 years of age), considering the low volume of vitreous fluid in them.

Considering the short duration of the study and the nonavailability of adequate literature on the same topic in the described geographic region (geographic region affects ambient temperatures and the potassium values of the vitreous indirectly), no specific sampling technique was employed for the study. All deceased individuals who did not fall under the exclusion criteria and met the inclusion criteria were included in the study. Using a sterile 20-gauge needle, samples of vitreous humor were aspirated progressively and slowly from the posterior chamber through a puncture 5-6 mm distant from the limbus (ora serrata). A sample between 1 and 5 ml was taken from each eye. As vigorous removal or the use of vacuum tubes could cause dislodged retinal tissue and contamination, care was taken to prevent tearing any loose tissue pieces surrounding the vitreous chamber [12-15]. Turbid blood-filled samples were discarded. For the purpose of completing the technique on cadaveric specimens, the study's primary investigator received preparatory simulation training; nevertheless, those samples were not used in the study. The obtained samples of vitreous humor were immediately sent to the Central Laboratory, Department of Biochemistry, at our hospital for analysis of vitreous potassium levels with the help of an automated electrolyte analyzer (Accu-Life, Compact Diagnostics India Pvt. Ltd., New Delhi, India).

Additionally, details about the time of death were acquired from police files, hospital records, or eyewitnesses, kin, friends, and other close associates of the deceased. TSD as on police record was based on the doctor who has declared the death or the relative/person accompanying the deceased at the time of death. The TSD determined in this way was then double-checked using postmortem alterations such as rigor mortis. The formula proposed by Sturner and Gantner (1964) [16] was applied as follows: PMI (hrs) = 7.14 K+ (mEq/L) - 39.1, where PMI stands for PMI, and K+ stands for vitreous tumor potassium concentration.

The data were entered in MS Excel 2020 (Microsoft Corporation, Redmond, Washington) and were analyzed using the Statistical Package for the Social Sciences (SPSS) software, version 20 (IBM Corp., Armonk, NY). Categorical variables are expressed as the mean ± standard deviation and percentages. Comparative data are expressed as ratios and proportions. Spearman’s rho was used to establish the correlation between PMIs calculated from potassium values, PMIs estimated using physical signs, and PMIs determined using official police records.

## Results

A total of 100 deceased individuals were included in the study, and samples of their vitreous humor were analyzed for potassium levels. The baseline characteristics of the study population are given in Table 1.

**Table 1:** Baseline characteristics of the study population (n = 100)

Parameters	Category	Frequency (%)
Age	13-22 years	6 (6)
	23-32 years	15 (15)
	33-42 years	15 (15)
	43-52 years	23 (23)
	53-62 years	24 (24)
	63-72 years	14 (14)
	73-82 years	3 (3)
	>82 years	0 (0)
Gender	Male	68 (68)
	Female	32 (32)
Cause of death	Poisoning	4 (4)
	Trauma	18 (18)
	Asphyxia	33 (33)
	Unclassified	45 (45)

The study population was predominated by men (68%) and deceased individuals belonging to the age group of 53-62 years (24%). The most common cause of death was unclassified deaths (45%) followed by asphyxia deaths (33%). Table 2 shows the basic distribution of the PMI as calculated by physical signs (rigor mortis) from the official police records and the potassium levels.

**Table 2:** Data on postmortem interval (PMI) determined using potassium values in the vitreous humor, police official records, and physical signs (rigor mortis)

Parameters	PMI using potassium values	PMI using police official records	PMI using rigor mortis	Ambient temperature
Mean ± standard deviation	14.7 ± 8.62	14.0 ± 8.39	14.2 ± 8.53	31.5 ± 2.51
Median	13.7	15	13.2	31
Interquartile range	(7.3, 21.6)	(6, 20)	(6.8, 19.6)	(30, 33)

It also displays data on the available ambient temperature exerting a potential impact on the determination of TSD. Furthermore, no correlation was observed between the ambient temperature (temperature from where the body is recovered) and the potassium levels of the vitreous humor. Table 3 shows the summary of the correlation established between the PMIs estimated by police records and physical signs, the PMIs estimated by police records and potassium levels in vitreous humor, and the PMIs estimated by physical signs and potassium levels in vitreous humor individually using Spearman’s rho coefficient.

**Table 3:** Comparison of PMI values estimated using potassium in the vitreous humor, official records, and physical signs (rigor mortis) **Correlation is significant at the 0.01 level (two-tailed). PMI: Postmortem interval.

Correlations between PMI by physical signs and PMI as per records				
			PMI by physical signs	PMI from police official records
Spearman’s rho	PMI by physical signs (rigor mortis)	Correlation coefficient	1.000	0.901^**^
		Sig. (two-tailed)	-	0.000
		N	100	100
	PMI by police official records	Correlation coefficient	0.901^**^	1.000
		Sig. (two-tailed)	0.000	-
		N	100	100
Correlations between PMI as per records and PMI by potassium levels				
			PMI from police official records	PMI by potassium values in vitreous humor
Spearman’s rho	PMI from police official records	Correlation coefficient	1.000	0.943^**^
		Sig. (two-tailed)	-	0.000
		N	100	100
	PMI by potassium values in vitreous humor	Correlation coefficient	0.943^**^	1.000
		Sig. (two-tailed)	0.000	-
		N	100	100
Correlations between PMI by physical signs and PMI by potassium levels				
			PMI by physical signs (rigor mortis)	PMI by potassium values in vitreous humor
Spearman’s rho	PMI by physical signs (rigor mortis)	Correlation coefficient	1.000	0.872^**^
		Sig. (two-tailed)	-	0.000
		N	100	100
	PMI by potassium values in vitreous humor	Correlation coefficient	0.872^**^	1.000
		Sig. (two-tailed)	0.000	-
		N	100	100

**Figure 1:**
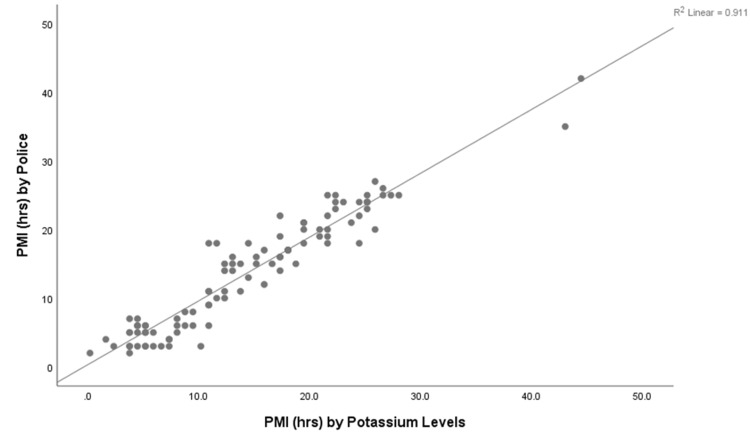
Scatter plot depicting the correlation between PMI derived from potassium values in vitreous and PMI from official police records PMI: Postmortem interval.

With all three findings being statistically significant at the two-tailed level or at the 0.01 level, there is not much of a change in the PMI calculated by each of the methods. Therefore, the PMI confirmed by the potassium levels distinguishes the PMI given by the police records and the physical signs. Additionally, Figure 1 (a scatter plot) and Table 4 depict the agreement between the PMIs estimated by the police records and the potassium values. The kappa value of 0.88 suggests an almost perfect agreement of PMI, given as per records, with that of the potassium levels. Based on the aforementioned findings, we can confirm that estimation of the TSD/PMI by calculating potassium levels is still a very good method as there is not much variation in the PMI when compared to PMI based on police records or physical signs, and the ambient temperature does not have much effect on the concentration of potassium in the vitreous humor.

**Table 4:** Agreement between PMI estimated by police official records and PMI derived from potassium values in the vitreous humor PMI: Postmortem interval.

Measure of agreement	Value	Asymptomatic standard error	Approximate T	Approximate significance
Kappa	0.880	0.044	10.886	0.0000
N (number of valid cases)				100

## Discussion

TSD estimation is essential in all criminal investigations. The body undergoes orderly changes following death as a result of physical, metabolic, autolytic, physiochemical, and biochemical processes, which can be used to estimate TSD [17]. A number of formulas have been developed over the last few years to calculate this interval using a combination of various statistical techniques and the concentrations of compounds found in vitreous humor [18]. In the current study, we looked at deceased people to determine whether vitreous potassium levels could be useful in determining PMI. The estimated TSD was adjusted for variables such as ambient temperature that can change the concentration of the substances found in the vitreous humor.

The current study proved that there is a positive linear relationship between PMI and vitreous potassium concentration. The potassium levels in the vitreous humor increased as the TSD increased (Spearman’s rho was statistically significant with values 0.901, 0.943, and 0.872 at the 0.01 or two-tailed level). These results support the findings of Cordeiro et al., Jashnani et al., James et al., Jaffe, Coe, Farmer et al., and Madea et al. [18-24]. Most of these studies proposed linear regression models. Additionally, an inverse correlation between the PMI and vitreous sodium/potassium ratio was discovered by Jashnani et al. While James et al. discovered that vitreous humor hypoxanthine levels can also be used to estimate the PMI, Cordeiro et al. suggested using vitreous hypoxanthine and urea levels to calculate the amount of TSD. His study also highlighted the use of software that could generate PMI from these electrolytes [18-20].

Age, sex, ambient temperature, and cause of death had no effect on the vitreous humor potassium values in the current study. Additionally, the TSD calculated using Sturner's suggested formula had a better correlation with the TSD as established by police records, the physical signs of rigor mortis, and other factors (kappa coefficient of 0.88). The results of our study are in line with those made by Jashnani et al. as well as Ahi and Garg, who claim that other factors such as sample age and sex had no bearing on the potassium levels in the vitreous humor [19,25].

Regarding the role of temperature in the levels of potassium concentration in the vitreous humor after death, this study discovered that there was no relationship between temperature and levels of potassium concentration. This finding is in line with the observations made by Sturner and Gantner, Jashnani et al., Jaffe, Ahi and Garg, Adelson et al., and Rao et al., who found that seasonal variation had little bearing on the rise in potassium levels following death [16,19,21,25-27].

Strengths and limitations

This study was exclusively conducted in cases of unnatural deaths along with a comparison of PMI determined by vitreous potassium with both physical signs of death and official police records, highlighting the accuracy of fluid electrolyte changes after death in determining PMI. Furthermore, this study contributes to the few available studies on the South Indian geographic population. The small sample size is one of the major limitations of the study. Owing to the short duration that TSD permits, the spectrum of the study was limited. Furthermore, there are plans to conduct a similar study on a larger population including all cases of death to determine the usefulness and association of vitreous potassium levels in determining PMI with various modes and causes of death in the future.

## Conclusions

Vitreous potassium concentration and TSD are correlated linearly. Age, gender, the cause of death, and the surrounding temperature have no discernible impact on the level of potassium in the vitreous. Additionally, there is a strong correlation between the estimated TSD determined by potassium levels in the vitreous humor and the actual TSD determined by rigor mortis and official police records. It has been discovered that using potassium measurements to estimate the PMI is linked to improved accuracy in all situations, making it a trustworthy marker for the same.

## References

[1] Aase S (2013). Autopsy--still the gold standard?. Tidsskr Nor Laegeforen.

[2] Henssge C, Madea B (2007). Estimation of the time since death. Forensic Sci Int.

[3] Madea B (2016). Methods for determining time of death. Forensic Sci Med Pathol.

[4] Reddy KSN, Murty OP (2014). The Essentials of Forensic Medicine and Toxicology. New Delhi: Jaypee Brothers Medical Publishers (P) Ltd.

[5] Gautam G (2015). Review of Forensic Medicine and Toxicology (Including Clinical & Pathological Aspects). Review of Forensic Medicine and Toxicology (Including Clinical & Pathological Aspects). 3rd ed. New Delhi: Jaypee Brothers Medical Publisher (P) Ltd.

[6] Ignatius PC (2018). Forensic Medicine and Toxicology. Forensic Medicine and Toxicology. 3rd ed. Kerala: Letterwave Books.

[7] Aggrawal A (2016). Forensic Medicine and Toxicology for MBBS. https://www.researchgate.net/publication/305498241_Forensic_Medicine_and_Toxicology_for_MBBS.

[8] Madea B (2015). Forensic Medicine: Findings, Reconstruction, Assessment (Book in German).

[9] Dell'Aquila M, De Matteis A, Scatena A, Costantino A, Camporeale MC, De Filippis A (2021). Estimation of the time of death: where we are now?. Clin Ter.

[10] Angayarkanni S (2021). Analysis of vitreous humour in determining postmortem interval (time since death) - a prospective study. Int J Forensic Med Toxicol Sci.

[11] Chand M, Kumar R, Anisha S, Saloni C (2019). Estimation of time since death by vitreous humor electrolytes concentration. Int J Forens Sci.

[12] Rognum TO, Holmen S, Musse MA, Dahlberg PS, Stray-Pedersen A, Saugstad OD, Opdal SH (2016). Estimation of time since death by vitreous humor hypoxanthine, potassium, and ambient temperature. Forensic Sci Int.

[13] Zilg B, Alkass K, Kronstrand R, Berg S, Druid H (2021). A rapid method for postmortem vitreous chemistry-deadside analysis. Biomolecules.

[14] Go A, Shim G, Park J, Hwang J, Nam M, Jeong H, Chung H (2019). Analysis of hypoxanthine and lactic acid levels in vitreous humor for the estimation of post-mortem interval (PMI) using LC-MS/MS. Forensic Sci Int.

[15] Madea B, Käferstein H, Hermann N, Sticht G (1994). Hypoxanthine in vitreous humor and cerebrospinal fluid--a marker of postmortem interval and prolonged (vital) hypoxia? Remarks also on hypoxanthine in SIDS. Forensic Sci Int.

[16] Sturner WQ, Gantner GE (1964). The postmortem interval: a study of potassium in the vitreous humor. Am J Clin Pathol.

[17] Ansari N, Menon SK (2017). Determination of time since death using vitreous humor tryptophan. J Forensic Sci.

[18] Cordeiro C, Ordóñez-Mayán L, Lendoiro E, Febrero-Bande M, Vieira DN, Muñoz-Barús JI (2019). A reliable method for estimating the postmortem interval from the biochemistry of the vitreous humor, temperature and body weight. Forensic Sci Int.

[19] Jashnani KD, Kale SA, Rupani AB (2010). Vitreous humor: biochemical constituents in estimation of postmortem interval. J Forensic Sci.

[20] James RA, Hoadley PA, Sampson BG (1997). Determination of postmortem interval by sampling vitreous humour. Am J Forensic Med Pathol.

[21] Jaffe FA (1962). Chemical post-mortem changes in the intraocular fluid. J Forensic Sci.

[22] Coe JI (1969). Postmortem chemistries on human vitreous humor. Am J Clin Pathol.

[23] Farmer JG, Benomran F, Watson AA, Harland WA (1985). Magnesium, potassium, sodium and calcium in post-mortem vitreous humour from humans. Forensic Sci Int.

[24] Madea B, Henssge C, Hönig W, Gerbracht A (1989). References for determining the time of death by potassium in vitreous humor. Forensic Sci Int.

[25] Ahi RS, Garg V (2011). Role of vitreous potassium level in estimating postmortem interval and the factors affecting it. J Clin Diagn Res.

[26] Adelson L, Sunshine I, Rushforth NB, Mankoff M (1963). Vitreous potassium concentration as an indicator of the postmortem interval. J Forensic Sci.

[27] Rao MNRB, Ravishankar R (2021). Estimation of time since death using vitreous humour potassium values. Indian J Forensic Med Toxicol.

